# TGFβ1 mimetic peptide modulates immune response to grass pollen allergens in mice

**DOI:** 10.1111/all.14108

**Published:** 2019-12-12

**Authors:** Galber R. Araujo, Lorenz Aglas, Emília R. Vaz, Yoan Machado, Sara Huber, Martin Himly, Albert Duschl, Luiz R. Goulart, Fatima Ferreira

**Affiliations:** ^1^ Department of Biosciences University of Salzburg Salzburg Austria; ^2^ Laboratory of Nanobiotechnology Institute of Biotechnology Federal University of Uberlândia Uberlândia Brazil; ^3^ Present address: Department of Oral Biological and Medical Sciences University of British Columbia Vancouver BC Canada

**Keywords:** allergic sensitization, grass pollen allergy, immune regulation, immunotherapy, TGFβ1

## Abstract

**Background:**

Transforming growth factor β1 (TGFβ1) is a cytokine that exerts immunosuppressive functions, as reflected by its ability to induce regulatory T (Treg) cell differentiation and inhibit Th1 and Th2 responses. Hence, peptides that mimic the active core domain of TGFβ1 may be promising candidates for modulation of the allergic response. This study aimed to investigate a synthetic TGFβ1 mimetic peptide (TGFβ1‐mim) for its ability to modulate the immune response during allergic sensitization to grass pollen allergens.

**Methods:**

The in vitro action of TGFβ1‐mim was evaluated in human lung epithelial cells, Jurkat cells, and rat basophilic leukemia cells. The in vivo action was evaluated in a murine model of Phl p 5 allergic sensitization. Additionally, the Th2 modulatory response was evaluated in IL‐4 reporter mice.

**Results:**

In vitro, TGFβ1‐mim downregulated TNF‐α production, IL‐8 gene expression, and cytokine secretion, upregulated IL‐10 secretion, and inhibited Phl p 5‐induced basophil degranulation. During Phl p 5 sensitization in mice, TGFβ1‐mim downregulated IL‐2, IL‐4, IL‐5, IL‐13, and IFN‐γ, upregulated IL‐10, and induced Treg cell production. Furthermore, mice treated with TGFβ1‐mim had lower levels of IgE, IgG1, IgG2a and higher levels of IgA antibodies than control mice. In a reporter mouse, the mimetic inhibited Th2 polarization.

**Conclusion:**

The TGFβ1‐mim efficiently modulated various important events that exacerbate the allergic microenvironment, including the production of main cytokines that promote Th1 and Th2 differentiation, and the induction of allergen‐specific regulatory T cells, highlighting its potential use in therapeutic approaches to modulate the immune response toward environmental allergens.

## Graphical abstract

TGFβ1‐mim downregulated major cytokines involved in Th1 and Th2 responses in mice sensitized to Phl p 5. Treatment of mice with the mimetic during sensitization to Phl p 5 induced Treg production, suppressed specific IgE and IgG1, IgE‐mediated basophil degranulation, induced IgA antibodies and IL‐10 secretion. In an IL‐4 reporter model, TGFβ1‐mim suppressed Th2 polarization induced by grass pollen extract.

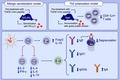

**Abbreviations**
4get: IL‐4/GFP‐enhanced transcript
Phl p: *Phleum pratense*
RBL cells: Rat basophilic leukemia cells
TGFβ1: Transforming growth factor β1
TGFβ1‐mim: TGFβ1 mimetic peptide
TGFβRII: TGFβ receptor II
Treg: Regulatory T cell

## 1 INTRODUCTION

Allergens are environmental proteins that interact with innate immune receptors, leading to Th2 polarization and consequently IgE production.^1^ Grasses are among the most potent sources of allergens. They produce large amounts of pollen, are widely distributed, and are potent respiratory allergens. Although it is estimated that only 8% of grass pollen‐allergic patients have been officially diagnosed, it is believed that around 20% of the population of the United States, Europe, and Brazil are affected by grass pollen allergy.^2^, ^3^ Hence, grass pollen allergy is a global problem, implying a need for novel therapeutic approaches. Almost all timothy grass (*Phleum pratense*) allergic patients displaying strong clinical reactions have high IgE antibody responses toward Phl p 5.^4^, ^5^, ^6^

Allergic sensitization is initiated when an allergen interacts with innate immune receptors, leading to Th2 polarization and IgE production. Upon re‐exposure, allergen cross‐links IgE bound to the high‐affinity FcεRI receptor on mast cells leading to inflammatory reactions characterized by the secretion of chemical mediators, synthesis of leukotrienes, prostaglandins, and cytokines (eg, IL‐4, IL‐5, and IL‐13), and by recruitment of other effector cells, such as Th17, Th9, basophils, and eosinophils.^7^, ^8^, ^9^ Th1 cells also contribute to the effector phase and chronicity in allergic diseases.

Regulatory T (Treg) cells are essential for immune tolerance against autoimmune and inflammatory diseases, allergies, and various other events related to the breakdown of immune homeostasis. Treg cells are a heterogeneous population characterized by the constitutive expression of the transcription factor Foxp3 and represent the dominant subset specific for common environmental allergens in healthy individuals.^10^, ^11^, ^12^ Transforming growth factor β1 (TGFβ1) is a key cytokine involved in the induction of Treg cells and in the regulation of effector T cells, B cells, and epithelial cells.^13^ In mice possessing a disrupted TGFβ1 gene or lacking the TGFβ receptor II (TGFβRII), severe inflammatory responses, tissue necrosis, and early death were observed,^14^, ^15^ confirming the broad immune regulatory functions of TGFβ1. Hence, the modulation of allergen‐specific CD4^+^ effector T cells by TGFβ1 could potentially induce immune tolerance and suppress allergic inflammation.

Considering that the availability, specificity, and biological activity of TGFβ1 are normally controlled by numerous interactions with membrane‐bound proteins,^16^ short peptides that mimic the binding domain of TGFβ1 and are able to efficiently activate the TGFβRII on target cells might represent novel therapeutic tools for treating allergies. We have previously selected by phage display a TGFβ1 mimetic peptide (pm26TGFβ1/TGFβ1‐mim) that was mapped into the TGFβ1:TGFβRII activation domain and shown to contain amino acid residues that are crucial for high‐affinity binding and stabilization. Furthermore, in the same study we also showed that the TGFβ1‐mim displayed potent anti‐inflammatory activity.^17^ Here, we investigated the ability of the TGFβ1‐mim to modulate the allergic/inflammatory response in vitro and in mice sensitized with Phl p 5 or timothy grass pollen extract. Our findings showed that the TGFβ1‐mim peptide efficiently modulated crucial events involved in allergen‐specific immune responses.

## 2 MATERIALS AND METHODS

### 2.1 Synthetic peptide

LPS‐free TGFβ1‐mim (pm26TGFβ1)^17^ was chemically synthesized by Bachem (Weil am Rhein, Germany). A detailed description is provided in the Appendix S1. Unconjugated human TGFβ1 recombinant protein produced in CHO cells (eBioscience, Austria) was included as positive control in the in vitro experiments.

### 2.2 IL‐8 expression analysis

To investigate whether TGFβ1‐mim affected IL‐8 expression, an A549 immortalized human lung epithelial cell line (A549 ‐ ATCC, LGC Promochem, Wesel, Germany) stably transfected with a luciferase reporter gene placed under the control of the IL‐8 promoter was used according to the original protocol.^18^ A detailed description is provided in the Appendix S1.

### 2.3 IL‐10 and TNF‐α measurements

Levels of secreted IL‐10 and TNF‐α in the supernatant of Jurkat cells and IL‐8 in the supernatant of A549 cells were measured by ELISA. Cells at a density of 4 × 10^5^/mL were pretreated with 1 µmol/L TGFβ1‐mim or recombinant TGFβ1 (rTGFβ1) for 1 hour and stimulated with 40 ng/mL phorbol 12‐myristate 13‐acetate (PMA) (Jurkat cells; Sigma‐Aldrich) plus 500 ng/mL ionomycin (Sigma‐Aldrich), or 20 ng/mL TNF‐α (A549 cells; GenScript) for 24 hours at 37°C and 5% CO_2_. Supernatant was collected for cytokine measurements, which were performed according to the manufacturer's protocol (eBioscience).

### 2.4 Mice sensitization model

Female BALB/c mice (6‐10 weeks old) were obtained from Charles River Laboratories and maintained in the animal facility of the University of Salzburg. To induce allergen‐specific IgE response, five mice were immunized intradermally (i.d.) with 25 µg of recombinant Phl p 5^19^ diluted in PBS without any added adjuvant. Five mice were pretreated subcutaneously with 100 µL of TGFβ1‐mim diluted in PBS at 1 µmol/L, followed by i.d. injection with 25 µg of Phl p 5 diluted in PBS. Three mice constituted the naïve group. Immunization was performed on days 0, 14, 28, and 42, and mice were sacrificed on day 44. To investigate the induction of Phl p 5‐specific IgE responses, blood samples were drawn from the saphenous vein on days 14, 28, and 42.

#### 2.4.1 In vivo Th2 polarization model

Bicistronic IL‐4 reporter mice in the BALB/c background (8‐13 weeks old) were obtained from The Jackson Laboratory. Because our preliminary data showed that rPhl p 5 alone did not induce IL‐4 production, we investigated the Th2 polarizing capacity of grass pollen extract alone or in combination with the mimetic. Five mice received i.d. injection in the right lateral abdominal region either with 25 µg rPhl p 5 (n = 5), 10 µg grass pollen extract (n = 5), or 10 µg grass pollen extract in combination with TGFβ1‐mim at 1 µmol/L diluted in PBS without added adjuvant. Five mice constituted the naïve group. Mice were sacrificed on the 5th day after allergen injection. IL‐4 expressing cells in skin‐draining inguinal lymph nodes were analyzed by flow cytometry. Following the principles of Russell and Burch's 3Rs^20^ for animal experiments, we analyzed the effects of the mimetic peptide on Phl p 5 responses without including recombinant TGFβ1. All animal experiments were performed according to national guidelines approved by the Austrian Federal Ministry of Science, Research and Economy (normal mice: BMWF‐66.012/0017‐WF/V/3b/2017; 4get mice: BMWF‐66.012/0041‐WF/V/3b/2017).

### 2.5 Rat basophil leukemia (RBL) cell mediator release assay

The capacity of TGFβ1‐mim to inhibit Phl p 5‐induced degranulation in murine RBL‐2H3 (muRBL) cells (ATCC^®^ CRL2256™) was performed as described elsewhere.^21^ Humanized RBL‐2H3 (huRBL) cells transfected with the cDNA encoding the human FcɛRI were tested with nine sera from grass pollen‐allergic patients, as described elsewhere.^22^ Experiments using anonymized human serum samples were approved by the local Ethics Committee of the Medical University and General Hospital of Vienna (no. EK1263/2014), and informed written consent was obtained from all study participants. A detailed description is provided in the Appendix S1.

### 2.6 Phl p 5‐specific antibody detection by ELISA

The levels of Phl p 5‐specific IgE, IgG1, IgG2a, and IgA antibodies were measured by ELISA in sera from all groups of mice. A detailed description is provided in the Appendix S1.

### 2.7 Cytokine‐producing cells detection by ELISPOT

Production of IFN‐γ, IL‐4, and IL‐10 from splenocytes in response to Phl p 5 stimulation was assessed by ELISPOT. A detailed description is provided in the Appendix S1.

### 2.8 Flow cytometry analysis

Flow cytometry analyses of proliferating mice splenocytes upon Phl p 5 stimulation for 5 days were performed as described in the Appendix S1. In this study, we investigated the percentage of allergen‐specific CD4^+^Foxp3^+^ Treg cells expressing CD25, GATA3, CTLA4, or the cellular proliferation marker KI67, important factors associated to Treg cell function.

### 2.9 Statistical analysis

Statistical analyses were conducted using GraphPad Prism 5 software (GraphPad software). Results are presented as mean with SD of each group. Comparisons between groups were performed using one‐way ANOVA for all experiments, except for the human RBL assays where the paired Student's *t* test was employed. *P* values < .05 (*P < .05; **P < .01; ***P < .001) were considered significant.

## 3 RESULTS

### 3.1 TGFβ1‐mim modulates cytokine production and IgE‐mediated basophil degranulation in vitro

**Figure 1:**
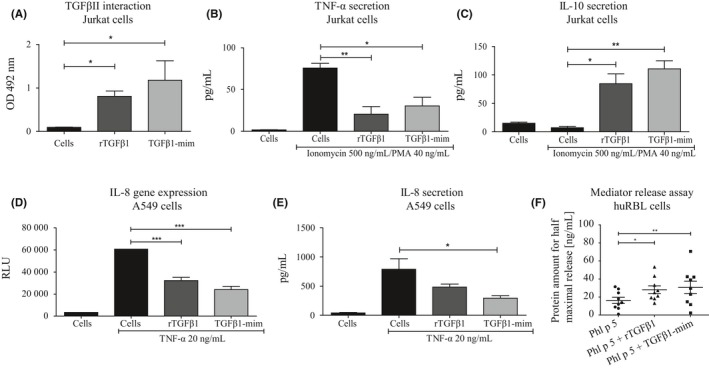
TGFβ1‐mim modulates immune responses in vitro. A, ELISA assay to assess binding of rTGFβ1 and TGFβ1‐mim to TGFβII on Jurkat cells. B, Secretion of TNF‐α and IL‐10 (C) in Ionomycin/PMA‐stimulated Jurkat cells. D, Luciferase reporter assay showing IL‐8 gene expression in TNF‐α‐stimulated A549 cells. E, IL‐8 secretion in TNF‐α‐stimulated A549 cells. F, Mediator release assay in huRBL cells expressing the human FcεRI. The ability of TGFβ1‐mim to suppress IgE‐mediated degranulation in sensitized basophils was confirmed in nine grass pollen‐sensitized patients. *P < .05, **P < .01, ***P < .001

We first tested the ability of the TGFβ1‐mim peptide to recognize TGFβRII on Jurkat cells, using ELISA. Recombinant TGFβ1 was used as a positive control. Although not statistically significant, TGFβ1‐mim showed slightly elevated reactivity to TGFβRII than rTGFβ1 (Figure 1A). In PMA‐stimulated Jurkat cells, both mimetic and rTGFβ1 significantly decreased the secretion of TNF‐α (Figure 1B) and increased the secretion of IL‐10 (Figure 1C). Since the lung epithelium plays an important role as a first line of defense toward external compounds,^18^ we sought to investigate the production of IL‐8 using a human lung epithelial (A549) cell line possessing a luciferase reporter gene under the control of the IL‐8 promoter. In TNF‐α‐stimulated A549 cells, both TGFβ1‐mim and rTGFβ1 decreased IL‐8 gene expression (Figure 1D), whereas only TGFβ1‐mim significantly decreased IL‐8 secretion (Figure 1E). To investigate whether the mimetic modulates IgE‐mediated basophil degranulation in an already established allergic microenvironment, we performed mediator release assays using RBL cells expressing the humanized FcεRI receptor. We found that the amount of Phl p 5 needed to induce half‐maximal release in basophils, which were passively sensitized with sera from grass pollen‐allergic patients, was significantly higher when cells were pretreated with TGFβ1‐mim (Figure 1F), indicating an suppression of degranulation. This effect was also observable when pretreating with rTGFβ1. Titration curves for every patient are presented in Figure E1. These results indicate that the TGFβ1‐mim was able to modulate the allergic inflammatory microenvironment in vitro.

### 3.2 TGFβ1‐mim modulates antibody response in vivo

**Figure 2:**
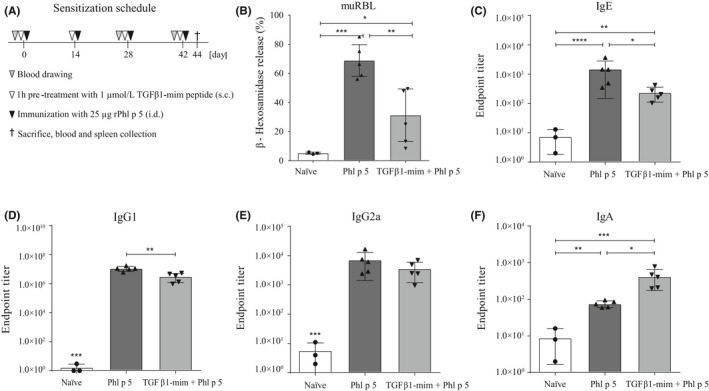
TGFβ1‐mim peptide modulates antibody response in vivo. A, Scheme of animal sensitization. B, Immunization with the recombinant Phl p 5 significantly induced IgE‐mediated basophil degranulation, whereas it was substantially decreased in mice that received the mimetic. TGFβ1‐mim peptide was able to suppress Phl p 5‐specific IgE (C), IgG1 (D) and IgG2a (E) and to induce IgA (F) antibody production in mice sera. *P < .05, **P < .01, ***P < .001. s.c., subcutaneous; i.d., intradermal

We next tested the ability of the TGFβ1‐mim to modulate the antibody response during Phl p 5 sensitization. Phl p 5 (Figure 2A) significantly induced allergen‐specific IgE response, as observed from the capacity of mice sera to provoke degranulation in RBL cells (Figure E2 and Figure 2B). RBL cells sensitized with sera of mice treated with 1 µmol/L TGFβ1‐mim had significantly lower levels of degranulation than the group injected with Phl p 5 alone. ELISA analysis showed that in serum from Phl p 5‐sensitized mice, TGFβ1‐mim significantly suppressed the levels of allergen‐specific IgE (Figure 2C) and IgG1 (Figure 2D). No significant differences were observed for allergen‐specific IgG2a antibodies (Figure 2E). Mice treated with TGFβ1‐mim also showed significant higher levels of allergen‐specific IgA (Figure 2F). Therefore, TGFβ1‐mim modulated the Phl p 5‐specific antibody responses in mice.

### 3.3 TGFβ1‐mim modulates cytokine production in vivo

**Figure 3:**
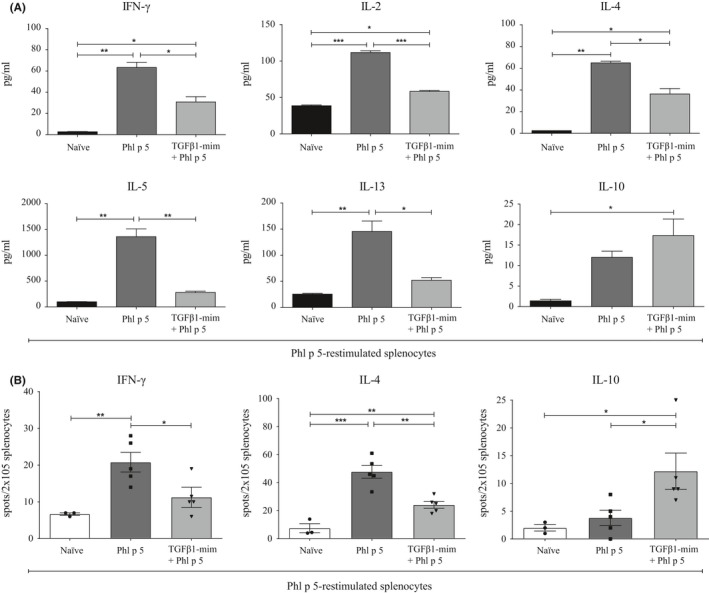
TGFβ1‐mim peptide modulates Th1 and Th2 responses in vivo. A, Supernatant from Phl p 5‐restimulated splenocytes assessed by multiplex bead‐based flow cytometry had significant reduction in the levels of secreted IFN‐γ, IL‐2, IL‐4, IL5 and IL‐13, and induction of IL‐10 in mice that received the TGFβ1‐mim peptide. Multiplex flow cytometry results were obtained on pooled samples. B, Phl p 5‐restimulated splenocytes assessed by ELISPOT rendered significant reduction in levels of secreted IFN‐γ and IL‐4, and induction of IL‐10 in mice that received the TGFβ1‐mim peptide. *P < .05, **P < .01, ***P < .001

To investigate the cytokine profile in the supernatants of restimulated splenocytes, we used a multiplex cytokine analysis kit. The secretion of IFN‐γ, IL‐2, IL‐4, IL‐5, and IL‐13 was significantly lower, whereas secretion of IL‐10 was higher in mice treated with TGFβ1‐mim than in untreated mice (Figure 3A). To determine the antigen‐specific T cell polarization upon TGFβ1‐mim treatment, the number of IFN‐γ, IL‐4, and IL‐10 producing splenocytes in all groups was assessed by ELISPOT. Sensitization with Phl p 5 resulted in high induction of IFN‐γ and IL‐4 release upon allergen stimulation. In mice treated with TGFβ1‐mim, levels of IFN‐γ and IL‐4 secreted by Phl p 5‐restimulated splenocytes were significantly lower, while levels of IL‐10 were significantly higher than in untreated mice (Figure 3B). Therefore, TGFβ1‐mim downregulated Th1 and Th2 cytokines, and upregulated IL‐10.

### 3.4 TGFβ1‐mim induces allergen‐specific Treg cell production

**Figure 4:**
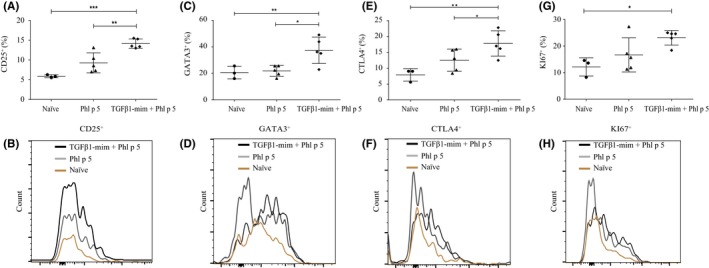
TGFβ1‐mim peptide induces Treg cell in vivo. Frequency of CD4 Foxp3 T cells expressing CD25 (A‐B), GATA3 (C‐D), CTLA4 (E‐F) and KI67 (G‐H) in splenocytes was enriched in mice that received the TGFβ1‐mim peptide. *P < .05, **P < .01, ***P < .001

To investigate the influence of TGFβ1‐mim on Phl p 5‐specific Treg induction during allergic sensitization, we analyzed splenocytes from BALB/c mice using flow cytometry. We sought to investigate CD4^+^Foxp3^+^ cells expressing CD25, GATA3, CTLA4, or KI67, since these factors are required for Treg cell survival and function.^23^, ^24^ The percentage of CD4^+^CD25^+^Foxp3^+^ proliferating Treg cells found in naïve and Phl p 5‐sensitized mice was 5.81 ± 0.4 and 8.5 ± 2.5, respectively, whereas 13.5 ± 1.1 was found in mice treated with TGFβ1‐mim (Figure 4A‐B). Naïve and Phl p 5‐sensitized mice had comparable levels of CD4^+^Foxp3^+^ Treg cells expressing GATA3 (20.2 ± 4.7% and 22.4 ± 4%, respectively), whereas mice treated with TGFβ1‐mim had 36.2 ± 10% (Figure 4C‐D). The percentage of CD4^+^Foxp3^+^ Treg cells expressing CTLA4 in naïve and Phl p 5‐sensitized mice was 9.0 ± 1.9% and 13.1 ± 3.5%, respectively, whereas for mice treated with TGFβ1‐mim this value was 17.5 ± 4.0% (Figure 4E‐F). Although not significantly different when compared to Phl p 5‐sensitized mice (15.7 ± 6.4%), the percentage of CD4^+^Foxp3^+^ Treg cells expressing the KI67 proliferation marker was enriched in the group of mice treated with TGFβ1‐mim (24.6 ± 2.8) (Figure 4G‐H). These results demonstrate that TGFβ1‐mim promotes Treg cell production during Phl p 5 sensitization.

### 3.5 TGFβ1‐mim suppresses Th2 polarization in vivo

**Figure 5:**
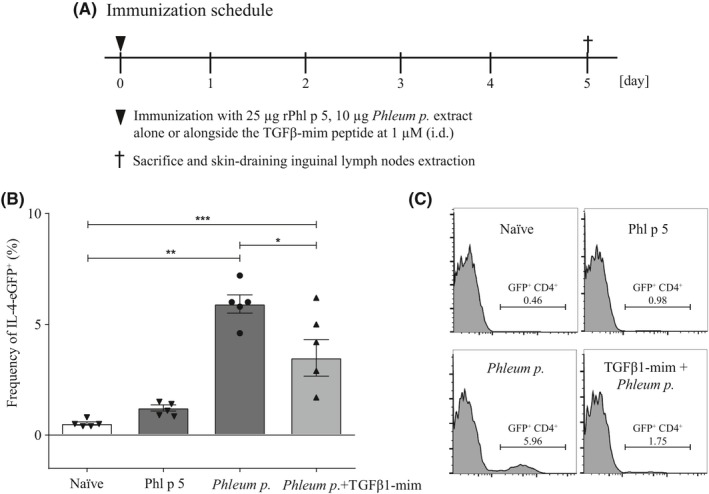
TGFβ1‐mim peptide inhibits Th2 polarization in IL‐4 reporter mouse model. (A) 4get mice were sacrificed 5 days after allergen injection to analyze the frequency of CD4+ EGFP+ expressing skindraining inguinal lymph node cells. (B‐C) TGFβ1‐mim administered alongside the grass pollen extract was able to downregulate Th2 polarization. Phl p 5 by itself was not able to induce a significant Th2 polarization. *P < .05, **P < .01, ***P < .001. i.d., intradermal

Th2 lymphocytes produce the cytokines IL‐4, IL‐5, and IL‐13, and drive the class switching toward IgE in B cells.^25^ To investigate whether the TGFβ1‐mim modulated Th2 polarization in the presence of grass pollen extract or rPhl p 5, we used an IL‐4 reporter mouse. The 4get mice express eGFP as part of a bicistronic IL‐4‐IRES‐GFP mRNA, allowing the identification of IL‐4 expressing cells *in vivo*.^26^, ^27^ Flow cytometry analysis of eGFP^+^CD4^+^ lymphocytes isolated from skin‐draining inguinal lymph node cells of 4get mice 5 days after allergen injection (Figure 5A) showed that TGFβ1‐mim administered alongside the grass pollen extract suppressed Th2 polarization (Figure 5B‐C). Phl p 5 injected alone did not induce Th2 polarization. In summary, the TGFβ1‐mim administered in combination with grass pollen extract was able to inhibit Th2 polarization in vivo.

## 4 DISCUSSION

For decades the Th1/Th2 balance has been the predominant concept for a healthy immune system. Accordingly, allergy has been proposed to result from a shift in the Th1/Th2 balance in favor of a Th2‐biased response. In this respect, activated Th2 lymphocytes produce IL‐4, IL‐5, and IL‐13, subsequently inducing B cells to produce allergen‐specific IgE antibodies. More recently, there has been increasing evidence that other T helper cell subsets such as Th9, Th17, and Th22 participate in the progression of certain forms of T cell‐mediated allergic disorders, suggesting further layers of complexity.^28^, ^29^, ^30^ In addition, extensive data have reported the regulatory effect of antigen‐specific Treg cells in the suppression of allergic responses.^31^, ^32^, ^33^, ^34^ Therefore, approaches aiming at the induction of Treg cells could modulate cytokine production, T cell polarization, and antibody profiles, all shown to be key events in inflammation and allergy. Here, we investigated the immunoregulatory action of a TGFβ1 mimetic peptide in vitro and in mice sensitized either with Phl p 5 or grass pollen extract.

IL‐8 is a chemoattractant cytokine produced by a variety of tissue and blood cells that predominantly attract neutrophils to inflammatory sites.^35^, ^36^ It has been shown that activated neutrophils accumulate at the site of allergic provocation and have the ability to prime CD4^+^ T cells,^37^ a crucial event for the initiation of allergic inflammation. Grass pollen extract can induce neutrophil immune responses through the secretion of IL‐8,^38^, ^39^ reinforcing the importance of this cytokine in the allergic response. We showed here that in TNF‐α‐stimulated lung epithelial cells, TGFβ1‐mim suppressed IL‐8 gene expression and cytokine secretion. Based on these results, we suggest that the reduction of neutrophil migration by TGFβ1‐mim previously described in a mouse model of peritonitis^17^ could be explained by its suppressive effects on IL‐8 secretion. Similarly, TGFβ1 has been shown to inhibit IL‐8 production.^40^, ^41^

In our murine in vivo model, sensitization was induced by Phl p 5. The pretreatment with TGFβ1‐mim followed by Phl p 5 sensitization resulted in the modulation of various events related to the exacerbation of the allergic response. Firstly, in vivo treatment with TGFβ1‐mim significantly inhibited antigen‐specific IgE and IgG1 antibody production and suppressed degranulation in basophils passively sensitized with sera from Phl p 5‐sensitized mice. TGFβ1‐mim enhanced IgA production, consistent with other studies reporting TGFβ1 as an IgA‐specific class‐switching factor.^42^, ^43^, ^44^ Furthermore, allergic disorders appear to be more common among patients with deficiency for IgA.^45^, ^46^ We also observed that sera from mice treated with TGFβ1‐mim had enhanced IgE‐allergen blocking capacity (data not shown), which will be further investigated in future studies.

TGFβ1‐mim efficiently modulated IFN‐γ and IL‐4 cytokine production in Phl p‐5‐restimulated splenocytes. Since IFN‐γ and IL‐4 are marker cytokines for Th1 and Th2 cells, respectively, and play major roles in the development and regulation of immune responses,^47^ the suppression of these two cytokines strongly suggests that the TGFβ1‐mim treatment suppressed the allergen‐induced inflammatory response. In parallel, the TGFβ1‐mim treatment significantly increased the number of IL‐10‐secreting cells. IL‐10 downregulates the development of cell‐mediated immune responses. It has been previously shown in a murine asthma model that the suppression of allergic airway disease correlates with an increase of IL‐10^+^CD4^+^ cells.^48^, ^49^ In addition, Phl p‐5‐restimulated splenocytes from mice treated with TGFβ1‐mim showed reduced levels of secreted IFN‐γ, IL‐2, IL‐4, IL‐5, and IL‐13 cytokines, which act in concert to determine T helper polarization.^10^

The induction of peripheral T cell tolerance and production of Treg cells are key mechanisms in allergen‐specific immunotherapy.^34^, ^50^ T cell‐specific deletion of TGFβRII in mice showed that TGFβ signaling is required for the maintenance and survival of peripheral Foxp3‐expressing Treg cells.^51^ In addition, treatment with TGFβ1 induces Foxp3 expression.^17^, ^52^, ^53^ Here, we found that mice administration of TGFβ1‐mim during Phl p 5 sensitization induced CD4^+^Foxp3^+^ Treg cells expressing CD25, GATA3, CTLA4, and the proliferation‐associated nuclear antigen KI67, important factors required for Treg cell survival and function. Treg cells characterized by the expression of CD4, CD25, and Foxp3 are a subpopulation of CD4^+^ T cells specialized to modulate the immune system, maintain immune tolerance, and prevent autoimmunity.^34^, ^54^, ^55^ Intrinsic expression of GATA3 controls Treg cell polarization and maintains Treg cell Foxp3 expression during inflammation in mice.^56^ CTLA‐4 is an inhibitory receptor crucial for the suppressive function of Treg cells in vivo. A specific deficiency of CTLA‐4 in Treg cells results in spontaneous development of systemic lymphoproliferation, lethal T cell‐mediated autoimmune disease, and hyperproduction of IgE in mice.^57^ Since the balance between allergen‐specific Treg cells and Th2 seems to play a decisive role in the development of the immune response to allergens,^58^ we hypothesize that the production of Treg cell induced by the TGFβ1‐mim in Phl p 5‐sensitized mice suppressed Th2 polarization. It should be pointed out that besides the herein described Treg phenotypes, there are additional Treg‐specific markers recently described allowing for a more thoroughly characterization of Treg subsets known to be associated with TGFβ and the induction of allergen tolerance. These include the transcription factor Helios, which is expressed in a large subset of Foxp3^+^ Tregs, the activation‐dependent surface marker TIGIT, and the leukocyte differentiation antigen CD226.^34^, ^59^, ^60^ The capacity of TGFβ1‐mim to induce Treg cells will be further explored in future studies.

In order to investigate the modulatory capacity of the TGFβ1‐mim on Th2 polarization in vivo, we used the 4get mouse model, which possesses a bicistronic mRNA linking a readily identifiable reporter (eGFP) to the IL‐4 gene expression.^26^, ^27^, ^61^, ^62^ In this model, recombinant Phl p 5 alone was not able to induce expression of eGFP in CD4^+^ T cells. In a recent study, we could show that birch pollen extracts promoted Th2 polarization in the 4get mouse model whereas purified recombinant Bet v 1 did not. These findings led us to hypothesize that the context in which an allergen is presented to the immune system plays a crucial role in the outcome of the allergen‐specific immune response.^27^ In line with these observations, here we showed that grass pollen extract induced Th2 polarization in the 4get mice but not rPhl p 5, thus reinforcing the idea that the context (adjuvants) provided by the pollen is crucial in the initiation of the Th2 polarization.

TGF‐β1 has been implicated in the pathogenesis of human fibrosis.^63^ Additionally, several cytokines including IFN‐γ, IL‐4, and IL‐13 have been shown to participate in the progression of fibrosis.^64^, ^65^, ^66^ A crucial role of toll‐like receptor 4 (TLR4) in fibrosis has also been suggested.^67^ Although the implication of the TGFβ1‐mim in the induction of fibrosis was not directly addressed in this study, we found that under in vivo inflammatory conditions induced by allergen sensitization, the mimetic was able to efficiently downregulate the production of IFN‐γ, IL‐4, and IL‐13 (Figure 3). We also observed that the mimetic was able to downregulate the LPS‐induced TLR4 expression in a human TL4 reporter cell line (data not shown). In this respect, overexpression of TLR4 after LPS challenge in mice or mouse lung fibroblasts was shown to significantly contribute to the induction of pulmonary fibrosis.^68^, ^69^ Taken together, we speculate that the ability to inhibit crucial players (IFN‐γ, IL‐4, and IL‐13 cytokines; TLR4 expression) involved in the induction of fibrosis rather supports the idea of a beneficial regulatory property for the mimetic peptide. Nevertheless, the possible role of the mimetic in fibrosis needs to be fully investigated before clinical applications in humans.

In summary, we have shown a regulatory role of a TGFβ1 mimetic peptide in the modulation of the inflammatory allergic response. TGFβ1‐mim regulated Th1 and Th2 responses *via* the modulation of cytokines and antibodies, induced Treg cell differentiation, and inhibited basophil degranulation. We propose that the application of TGFβ1 mimetic peptide prior to allergen vaccination might enhance the efficacy of conventional AIT protocols by modulating the allergic inflammation and increasing tolerance to the sensitizing allergen. Future studies should explore the immunomodulatory potential of the TGFβ1‐mim within other models of allergic sensitization (eg, food and house dust mite) as well as different routes of application (eg, mucosal and transdermal).

## CONFLICT OF INTEREST

F. Ferreira is a member of Scientific Advisory Boards (HAL Allergy, NL; SIAF, Davos, CH; AllergenOnline, USA). The remaining authors declare that they have no relevant conflicts of interest.

## AUTHOR CONTRIBUTIONS

GRA and LA performed immunoassays, cell culture, in vivo experiments and analysis. YM performed in vivo experiments. ERV performed RBL experiments. SH performed IgE‐allergen blocking capacity analyses based on the IgE‐facilitated allergen binding (FAB) assay. MH analyzed IL‐8 expression on A549 cells. GRA, LA, AD, LRG, and FF wrote the manuscript. FF supervised all aspects of the study. All authors read and approved the manuscript.

## References

[1] van Ree R , Hummelshoj L , Plantinga M , Poulsen LK , Swindle E . Allergic sensitization: host‐immune factors. Clin Transl Allergy. 2014;4(1):12. 24735802 10.1186/2045-7022-4-12 PMC3989850

[2] Garcia‐Mozo H . Poaceae pollen as the leading aeroallergen worldwide: a review. Allergy. 2017;72(12):1849‐1858. 28543717 10.1111/all.13210

[3] Kleine‐Tebbe J , Davies J . Grass pollen allergens In: Akdis CA, Agache I, eds. Global atlas of allergy. Zürich, Switzerland: European Academy of Allergy and Clinical Immunology (EAACI); 2014:22‐26.

[4] Sterner T , Uldahl A , Svensson A , et al. IgE sensitization in a cohort of adolescents in southern Sweden and its relation to allergic symptoms. Clin Mol Allergy. 2019;17:6. 30983886 10.1186/s12948-019-0110-6 PMC6444864

[5] Gobl C , Focke‐Tejkl M , Najafi N , et al. Flexible IgE epitope‐containing domains of Phl p 5 cause high allergenic activity. J Allergy Clin Immunol. 2017;140(4):1187‐1191. 28532654 10.1016/j.jaci.2017.05.005 PMC5632575

[6] Almeida E , Caeiro E , Todo‐Bom A , Duarte A , Gazarini L . Sensitization to grass allergens: Phl p1, Phl p5 and Phl p7 Phl p12 in adult and children patients in Beja (Southern Portugal). Allergol Immunopathol. 2019;47(6):579‐584. 10.1016/j.aller.2019.04.006 31477404

[7] Zhu J . T helper 2 (Th2) cell differentiation, type 2 innate lymphoid cell (ILC2) development and regulation of interleukin‐4 (IL‐4) and IL‐13 production. Cytokine. 2015;75(1):14‐24. 26044597 10.1016/j.cyto.2015.05.010 PMC4532589

[8] Koch S , Sopel N , Finotto S . Th9 and other IL‐9‐producing cells in allergic asthma. Semin Immunopathol. 2017;39(1):55‐68. 27858144 10.1007/s00281-016-0601-1

[9] Janeway AC , Travers P , Walport M , Shlomchik M . Immunobiology: The Immune System in Health & Disease, (5th ed). New York, NY: Garland Science; 2001.

[10] Berker M , Frank LJ , Gessner AL , et al. Allergies‐A T cells perspective in the era beyond the TH1/TH2 paradigm. Clin Immunol. 2017;174:73‐83. 27847316 10.1016/j.clim.2016.11.001

[11] Esensten JH , Muller YD , Bluestone JA , Tang Q . Regulatory T‐cell therapy for autoimmune and autoinflammatory diseases: the next frontier. J Allergy Clin Immunol. 2018;142(6):1710‐1718. 30367909 10.1016/j.jaci.2018.10.015

[12] Noval Rivas M , Chatila TA . Regulatory T cells in allergic diseases. J Allergy Clin Immunol. 2016;138(3):639‐652. 27596705 10.1016/j.jaci.2016.06.003 PMC5023156

[13] Troncone E , Marafini I , Stolfi C , Monteleone G . Transforming growth factor‐beta1/Smad7 in intestinal immunity, inflammation, and cancer. Front Immunol. 2018;9:1407. 29973939 10.3389/fimmu.2018.01407 PMC6019438

[14] Shull MM , Ormsby I , Kier AB , et al. Targeted disruption of the mouse transforming growth factor‐beta 1 gene results in multifocal inflammatory disease. Nature. 1992;359(6397):693‐699. 1436033 10.1038/359693a0 PMC3889166

[15] Marie JC , Liggitt D , Rudensky AY . Cellular mechanisms of fatal early‐onset autoimmunity in mice with the T cell‐specific targeting of transforming growth factor‐beta receptor. Immunity. 2006;25(3):441‐454. 16973387 10.1016/j.immuni.2006.07.012

[16] Weiss A , Attisano L . The TGFbeta superfamily signaling pathway. Wiley Interdiscip Rev Dev Biol. 2013;2(1):47‐63. 23799630 10.1002/wdev.86

[17] Vaz ER , Fujimura PT , Araujo GR , et al. A Short peptide that mimics the binding domain of TGF‐beta1 presents potent anti‐inflammatory activity. PLoS ONE. 2015;10(8):e0136116. 26312490 10.1371/journal.pone.0136116 PMC4552549

[18] Oostingh GJ , Schmittner M , Ehart AK , Tischler U , Duschl A . A high‐throughput screening method based on stably transformed human cells was used to determine the immunotoxic effects of fluoranthene and other PAHs. Toxicol in Vitro. 2008;22(5):1301‐1310. 18434080 10.1016/j.tiv.2008.03.003

[19] Kurtaj A , Hillebrand C , Fichtinger G , et al. Natural protective immunity against grass pollen allergy is maintained by a diverse spectrum of response types. J Allergy Clin Immunol. 2017;140(6):1746‐1749. 28867457 10.1016/j.jaci.2017.07.030

[20] Russell WMS , Burch RL . The Principles of Humane Experimental Technique. London, UK: Methuen; 1959.

[21] Wallmann J , Proell M , Stepanoska T , et al. A mimotope gene encoding the major IgE epitope of allergen Phl p 5 for epitope‐specific immunization. Immunol Lett. 2009;122(1):68‐75. 19111573 10.1016/j.imlet.2008.12.002 PMC2999763

[22] Vogel L , Luttkopf D , Hatahet L , Haustein D , Vieths S . Development of a functional in vitro assay as a novel tool for the standardization of allergen extracts in the human system. Allergy. 2005;60(8):1021‐1028. 15969682 10.1111/j.1398-9995.2005.00803.x

[23] Wang Y , Su MA , Wan YY . An essential role of the transcription factor GATA‐3 for the function of regulatory T cells. Immunity. 2011;35(3):337‐348. 21924928 10.1016/j.immuni.2011.08.012 PMC3182399

[24] Duhen T , Duhen R , Lanzavecchia A , Sallusto F , Campbell DJ . Functionally distinct subsets of human FOXP3+ Treg cells that phenotypically mirror effector Th cells. Blood. 2012;119(19):4430‐4440. 22438251 10.1182/blood-2011-11-392324 PMC3362361

[25] Kool M , Hammad H , Lambrecht BN . Cellular networks controlling Th2 polarization in allergy and immunity. F1000 Biol Rep. 2012;4:6. 22403589 10.3410/B4-6 PMC3292286

[26] Mohrs M , Shinkai K , Mohrs K , Locksley RM . Analysis of type 2 immunity in vivo with a bicistronic IL‐4 reporter. Immunity. 2001;15(2):303‐311. 11520464 10.1016/s1074-7613(01)00186-8

[27] Aglas L , Gilles S , Bauer R , et al. Context matters: Th2 polarization resulting from pollen composition and not from protein‐intrinsic allergenicity. J Allergy Clin Immunol. 2018;142(3):984‐987. 29782896 10.1016/j.jaci.2018.05.004 PMC6129402

[28] Sehra S , Yao W , Nguyen ET , et al. TH9 cells are required for tissue mast cell accumulation during allergic inflammation. J Allergy Clin Immunol. 2015;136(2):433‐440. 25746972 10.1016/j.jaci.2015.01.021 PMC4530056

[29] Choy DF , Hart KM , Borthwick LA , et al. TH2 and TH17 inflammatory pathways are reciprocally regulated in asthma. Sci Transl Med. 2015;7(301):301ra129. 10.1126/scitranslmed.aab3142 26290411

[30] Brunner PM , Pavel AB , Khattri S , et al. Baseline IL‐22 expression in patients with atopic dermatitis stratifies tissue responses to fezakinumab. J Allergy Clin Immunol. 2019;143(1):142‐154. 30121291 10.1016/j.jaci.2018.07.028

[31] Van Eden W , Van Der Zee R , Van Kooten P , et al. Balancing the immune system: Th1 and Th2. Ann Rheum Dis. 2002;61(Supplement 2):25ii‐28. 10.1136/ard.61.suppl_2.ii25 PMC1766722 12379616

[32] Dias S , D'Amico A , Cretney E , et al. Effector regulatory T Cell Differentiation and immune homeostasis depend on the transcription factor Myb. Immunity. 2017;46(1):78‐91. 28099866 10.1016/j.immuni.2016.12.017

[33] Kim BS , Kim IK , Park YJ , et al. Conversion of Th2 memory cells into Foxp3+ regulatory T cells suppressing Th2‐mediated allergic asthma. Proc Natl Acad Sci USA. 2010;107(19):8742‐8747. 20421479 10.1073/pnas.0911756107 PMC2889331

[34] Martin‐Orozco E , Norte‐Munoz M , Martinez‐Garcia J . Regulatory T Cells in allergy and asthma. Front Pediatr. 2017;5:117. 28589115 10.3389/fped.2017.00117 PMC5440567

[35] Admyre C , Axelsson LG , von Stein O , Zargari A . Immunomodulatory oligonucleotides inhibit neutrophil migration by decreasing the surface expression of interleukin‐8 and leukotriene B4 receptors. Immunology. 2015;144(2):206‐217. 25100544 10.1111/imm.12368 PMC4298415

[36] Bickel M . The role of interleukin‐8 in inflammation and mechanisms of regulation. J Periodontol. 1993;64(5):456‐460. 8315568

[37] Arebro J , Ekstedt S , Hjalmarsson E , Winqvist O , Kumlien Georen S , Cardell LO . A possible role for neutrophils in allergic rhinitis revealed after cellular subclassification. Sci Rep. 2017;7:43568. 28272395 10.1038/srep43568 PMC5341103

[38] Hosoki K , Boldogh I , Sur S . Innate responses to pollen allergens. Curr Opin Allergy Clin Immunol. 2015;15(1):79‐88. 25546327 10.1097/ACI.0000000000000136 PMC4361229

[39] Hosoki K , Itazawa T , Boldogh I , Sur S . Neutrophil recruitment by allergens contribute to allergic sensitization and allergic inflammation. Curr Opin Allergy Clin Immunol. 2016;16(1):45‐50. 26694038 10.1097/ACI.0000000000000231 PMC4941617

[40] Smith WB , Noack L , KhewGoodall Y , Isenmann S , Vadas MA , Gamble JR . Transforming growth factor‐beta 1 inhibits the production of IL‐8 and the transmigration of neutrophils through activated endothelium. J Immunol. 1996;157(1):360‐368. 8683138

[41] Ge Q , Moir LM , Black JL , Oliver BG , Burgess JK . TGF beta 1 induces IL‐6 and Inhibits IL‐8 release in human bronchial epithelial cells: the role of Smad2/3. J Cell Physiol. 2010;225(3):846‐854. 20607798 10.1002/jcp.22295

[42] Sonoda E , Matsumoto R , Hitoshi Y , et al. Transforming growth factor beta induces IgA production and acts additively with interleukin 5 for IgA production. J Exp Med. 1989;170:1415‐1420. J Immunol. 2009;182(1):14–19. 19109128

[43] Kim PH , Kagnoff MF . Transforming Growth Factor‐Beta‐1 Is a Costimulator for Iga Production. J Immunol. 1990;144(9):3411‐3416. 2329276

[44] Austin AS , Haas KM , Naugler SM , Bajer AA , Garcia‐Tapia D , Estes DM . Identification and characterization of a novel regulatory factor: IgA‐inducing protein. J Immunol. 2003;171(3):1336‐1342. 12874223 10.4049/jimmunol.171.3.1336

[45] Kim WJ , Choi IS , Kim CS , Lee JH , Kang HW . Relationship between serum IgA level and allergy/asthma. Korean J Intern Med. 2017;32(1):137‐145. 27586868 10.3904/kjim.2014.160 PMC5214712

[46] Yazdani R , Azizi G , Abolhassani H , Aghamohammadi A . Selective IgA deficiency: epidemiology, pathogenesis, clinical phenotype, diagnosis, prognosis and management. Scand J Immunol. 2017;85(1):3‐12. 27763681 10.1111/sji.12499

[47] Kubo M . Innate and adaptive type 2 immunity in lung allergic inflammation. Immunol Rev. 2017;278(1):162‐172. 28658559 10.1111/imr.12557

[48] Coomes SM , Kannan Y , Pelly VS , et al. CD4(+) Th2 cells are directly regulated by IL‐10 during allergic airway inflammation. Mucosal Immunol. 2017;10(1):150‐161. 27166557 10.1038/mi.2016.47

[49] Wilson MS , Pesce JT , Ramalingam TR , Thompson RW , Cheever A , Wynn TA . Suppression of murine allergic airway disease by IL‐2:anti‐IL‐2 monoclonal antibody‐induced regulatory T cells. J Immunol. 2008;181(10):6942‐6954. 18981114 10.4049/jimmunol.181.10.6942 PMC2706157

[50] Bacher P , Heinrich F , Stervbo U , et al. Regulatory T cell specificity directs tolerance versus allergy against aeroantigens in humans. Cell. 2016;167(4):1067‐1078. 27773482 10.1016/j.cell.2016.09.050

[51] Li MO , Sanjabi S , Flavell RA . Transforming growth factor‐beta controls development, homeostasis, and tolerance of T cells by regulatory T cell‐dependent and ‐independent mechanisms. Immunity. 2006;25(3):455‐471. 16973386 10.1016/j.immuni.2006.07.011

[52] Chen W , Jin W , Hardegen N , et al. Conversion of peripheral CD4+CD25‐ naive T cells to CD4+CD25+ regulatory T cells by TGF‐beta induction of transcription factor Foxp3. J Exp Med. 2003;198(12):1875‐1886. 14676299 10.1084/jem.20030152 PMC2194145

[53] Wan YY , Flavell RA . Identifying Foxp3‐expressing suppressor T cells with a bicistronic reporter. Proc Natl Acad Sci USA. 2005;102(14):5126‐5131. 15795373 10.1073/pnas.0501701102 PMC556008

[54] Zhao H , Liao X , Kang Y . Tregs: where we are and what comes next? Front Immunol. 2017;8:1578. 29225597 10.3389/fimmu.2017.01578 PMC5705554

[55] Zhao ST , Wang CZ . Regulatory T cells and asthma. J Zhejiang Univ Sci B. 2018;19(9):663‐673. 30178633 10.1631/jzus.B1700346 PMC6137416

[56] Wohlfert EA , Grainger JR , Bouladoux N , et al. GATA3 controls Foxp3(+) regulatory T cell fate during inflammation in mice. J Clin Invest. 2011;121(11):4503‐4515. 21965331 10.1172/JCI57456 PMC3204837

[57] Wing K , Onishi Y , Prieto‐Martin P , et al. CTLA‐4 control over Foxp3+ regulatory T cell function. Science. 2008;322(5899):271‐275. 18845758 10.1126/science.1160062

[58] Jutel M , Akdis M , Blaser K , Akdis CA . Mechanisms of allergen specific immunotherapy ‐ T‐cell tolerance and more. Allergy. 2006;61(7):796‐807. 16792576 10.1111/j.1398-9995.2006.01175.x

[59] Thornton AM , Lu J , Korty PE , et al. Helios(+) and Helios(‐) Treg subpopulations are phenotypically and functionally distinct and express dissimilar TCR repertoires. Eur J Immunol. 2019;49(3):398‐412. 30620397 10.1002/eji.201847935 PMC6402968

[60] Fuhrman CA , Yeh WI , Seay HR , et al. Divergent phenotypes of human regulatory T cells expressing the receptors TIGIT and CD226. J Immunol. 2015;195(1):145‐155. 25994968 10.4049/jimmunol.1402381 PMC4475416

[61] Shinkai K , Mohrs M , Locksley RM . Helper T cells regulate type‐2 innate immunity in vivo. Nature. 2002;420(6917):825‐829. 12490951 10.1038/nature01202

[62] Vicetti Miguel RD , Quispe Calla NE , Dixon D , et al. IL‐4‐secreting eosinophils promote endometrial stromal cell proliferation and prevent Chlamydia‐induced upper genital tract damage. Proc Natl Acad Sci USA. 2017;114(33):E6892‐E6901. 28765368 10.1073/pnas.1621253114 PMC5565408

[63] Walton KL , Johnson KE , Harrison CA . Targeting TGF‐beta Mediated SMAD Signaling for the Prevention of Fibrosis. Front Pharmacol. 2017;8:461. 28769795 10.3389/fphar.2017.00461 PMC5509761

[64] King TE Jr , Albera C , Bradford WZ , et al. Effect of interferon gamma‐1b on survival in patients with idiopathic pulmonary fibrosis (INSPIRE): a multicentre, randomised, placebo‐controlled trial. Lancet. 2009;374(9685):222‐228. 19570573 10.1016/S0140-6736(09)60551-1

[65] Doucet C , Brouty‐Boye D , Pottin‐Clemenceau C , Canonica GW , Jasmin C , Azzarone B . Interleukin (IL) 4 and IL‐13 act on human lung fibroblasts. Implication in asthma. J Clin Invest. 1998;101(10):2129‐2139. 9593769 10.1172/JCI741 PMC508801

[66] Hashimoto S , Gon Y , Takeshita I , Maruoka S , Horie T . IL‐4 and IL‐13 induce myofibroblastic phenotype of human lung fibroblasts through c‐Jun NH2‐terminal kinase‐dependent pathway. J Allergy Clin Immunol. 2001;107(6):1001‐1008. 11398077 10.1067/mai.2001.114702

[67] Jiang D , Liang J , Fan J , et al. Regulation of lung injury and repair by Toll‐like receptors and hyaluronan. Nat Med. 2005;11(11):1173‐1179. 16244651 10.1038/nm1315

[68] He Z , Zhu Y , Jiang H . Inhibiting toll‐like receptor 4 signaling ameliorates pulmonary fibrosis during acute lung injury induced by lipopolysaccharide: an experimental study. Respir Res. 2009;10:126. 20017955 10.1186/1465-9921-10-126 PMC2803172

[69] He Z , Gao Y , Deng Y , et al. Lipopolysaccharide induces lung fibroblast proliferation through Toll‐like receptor 4 signaling and the phosphoinositide3‐kinase‐Akt pathway. PLoS ONE. 2012;7(4):e35926. 22563417 10.1371/journal.pone.0035926 PMC3338545

