# Supplementary material for: TGFβ1 mimetic peptide modulates immune response to grass pollen allergens in mice
Source: Allergy. 2019 Dec 12;75(4):882–91. doi: 10.1111/all.14108 (PMC7217028; doi:10.1111/all.14108)
Supplement: Supplementary file 1 [file ALL-75-882-s001.docx]

**Online repository for:**

**TGFβ1 mimetic peptide modulates immune response to grass pollen allergens in mice**

Galber R. Araujo PhD^1^, Lorenz Aglas PhD^1^, Emília R. Vaz MSc^2^, Yoan Machado PhD^1*^, Sara Huber MSc^1^, Martin Himly PhD^1^, Albert Duschl PhD^1^, Luiz R. Goulart PhD^2^, Fatima Ferreira PhD^1^

**Author information:** ^1^Department of Biosciences, University of Salzburg, Salzburg, Austria; ^2^Laboratory of Nanobiotechnology, Institute of Genetics and Biochemistry, Federal University of Uberlândia, Uberlândia, Brazil; *Present affiliation: Department of Oral Biological and Medical Sciences, University of British Columbia, Vancouver, Canada;

**Correspondence to:**Fatima Ferreira, PhD, Department of Biosciences, University of Salzburg, Hellbrunnerstr. 34, A-5020, Salzburg, Austria
Tel.: +43-662-8044-5016, Fax: +43-662-8044-745016; E-mail: [Fatima.ferreira@sbg.ac.at](mailto:Fatima.ferreira@sbg.ac.at)

**Word count:** 1862

**Methods**

**TGFβ1-mim peptide synthesis and detection on the surface of Jurkat cells**

The TGFβ1-mim peptide originally composed of seven amino acids was constructed with fourteen residues and contained two cysteine residues to induce disulfide bond formation and folding. Synthetic sequence: ACESPLKRQCGGGS (original peptide is underlined).

The capacity of the TGFβ1-mim peptide to recognize the TGFβ1 receptor on Jurkat cells (ATCC, LGC Promochem, Wesel, Germany) was evaluated by ELISA. Maxisorp microtiter 96-well plates (Nunc, Roskilde, Denmark) were coated with 1 × l0^6^ cells diluted in PBS, and incubated overnight at 4°C. Cells were blocked with 3% PBS-BSA for 1 hour at 37°C, washed once with PBS and incubated with either 1 µg/ml of the TGFβ1-mim peptide or 5ng/ml of recombinant TGFβ1 (eBioscience, Vienna, Austria) for one hour at 37°C. After washing, the plate was incubated for 1 h at 37°C with anti-human TGFβ (eBioscience, Vienna, Austria). Plate was washed and incubated with HRP-labeled anti-human IgG (Southern Biotech, Birmingham, USA) antibody for 1 h at 37°C. After washing, TMB substrate solution (Sigma, Vienna, Austria) was added to the plate, the reaction stopped by 2N H_2_SO_4_ solution and read in a microplate reader (Tecan reader infinite m200).

**IL-8 expression analysis**

A549 cells used in this experiment were derived from a lung epithelial carcinoma and produce a range of cytokines that are typical for epithelial cells upon suitable stimulation. The experiment was conducted according to the original protocol with modifications ^1^. These cells are cultured in RPMI 1640 without l-glutamine, supplemented with l-glutamine, penicillin/streptomycin, and 10% FCS. All cell culture reagents were obtained from PAA Laboratories (Pasching, Austria). A total of 4 × 10^5^/ml were cultured in the presence of G418 (0.5 mg/ml) overnight at 37°C and 5% CO_2_. Cells were pre-treated with 1µM TGFβ1-mim peptide or recombinant TGFβ1 for 1h, followed by addition of 20ng/ml TNF-α (GenScript, Piscataway, USA), or left untreated for 24 h at 37°C and 5% CO_2_. Supernatant was removed and 50 µl lysis buffer (100 mM potassium phosphate pH 7.8, 0.1% Triton, 1 mM DTT) was added to the cells and incubated for 10 min at 37°C and 5% CO_2_. Lysed cells were transferred to white 96-well microtiter plates and the luciferase signal was detected using a microplate reader (Tecan reader infinite m200) immediately after lysis by using a freshly prepared D-luciferin substrate (Synchem, Felsberg, Germay).

**RBL cell mediator release assay**

For mediator release assays, RBL-2H3 transfected with the alpha chain of the mouse or human IgE receptor were passively sensitized with IgE antibodies from Phlp 5-sensitized mice or grass pollen allergic patients, respectively, according as previously described ^2^ with modifications. a Briefly, a total of 4 × 10^4^ cells/well diluted in RPMI 1640 (supplemented with 10% (v/v) heat-inactivated fetal calf serum, 100 U penicillin and streptomycin/ml, 4 mM l-glutamine, 2 mM sodium pyruvate, 10 mM HEPES and 100 μM of 2-mercaptoethanol) were plated into 96-well tissue culture plates (Becton-Dickinson, Franklin Lakes, NJ, USA) and sensitized with individual sera from the different grass pollen allergic patients diluted at 1:20 by overnight incubation at 37°C and 7.5% CO_2_. Subsequently, cells were washed twice with Tyrode’s buffer (19 g/L Tyrode´s salt, 1 g/L NaHCO_3_, pH 7.2) supplemented with 0.1% BSA (Sigma-Aldrich, St. Louis, MO, USA) and incubated with the TGFβ1-mim peptide diluted at 1µM for 24 hours at 37°C and 7.5% CO_2_. For allergen stimulation, cross-linking of FcɛRI-bound IgE was induced by addition of Phl p 5 diluted in Tyrode’s buffer in 1:10 dilutions starting at 1µM/ml for 1 hour at 37°C and 7.5% CO_2_. For maximal β-hexosaminidase release, 10 μl 10% Triton X-100 was added to the wells containing cells only. Thereafter, 50 μl of supernatants was transferred to a 96-well plate (Greiner) and incubated with 50 μl 4-Methylumbelliferyl N-acetyl-β-D-glucosaminide (4-MUG) (Sigma, Deisenhofen, Germany) in citrate buffer (0.1 M, pH 4.5) for 1 hour at conditions indicated above. The reaction was stopped by adding 100 μl glycine buffer (0.2 M glycine and 0.2 M NaCl, pH 10.7) and fluorescence was measured at 465–360 nm using a microplate reader (Tecan reader infinite m200). For muRBL assay, a total of 4 × 10^4^ cells/well diluted in RPMI 1640 (supplemented with 10% (v/v) heat-inactivated fetal calf serum, 100 U penicillin and streptomycin/ml, 4 mM l-glutamine, 2 mM sodium pyruvate, 10 mM HEPES and 100 μM of 2-mercaptoethanol) were plated into 96-well tissue culture plates (Becton-Dickinson, Franklin Lakes, NJ, USA) and sensitized with individual sera from the different immunization groups or naïve mice at a final dilution of 1:20 by overnight incubation at 37°C and 7.5% CO_2_. Subsequently, cells were washed twice with Tyrode’s buffer (19 g/L Tyrode´s salt, 1 g/L NaHCO_3_, pH 7.2) supplemented with 0.1% BSA (Sigma-Aldrich, St. Louis, MO, USA). For stimulation, cross-linking of FcɛRI-bound IgE was induced by addition of Phl p 5 diluted at 1ng/ml in Tyrode’s buffer for 1 hour at 37°C and 7.5% CO_2_. For maximal β-hexosaminidase release, 10 μl 10% Triton X-100 was added to the wells containing cells only. Thereafter, 50 μl of supernatants was transferred to a 96-well plate (Greiner) and incubated with 50 μl 4-Methylumbelliferyl N-acetyl-β-D-glucosaminide (4-MUG) (Sigma, Deisenhofen, Germany) in citrate buffer (0.1 M, pH 4.5) for 1 hour at conditions indicated above. The reaction was stopped by adding 100 μl glycine buffer (0.2 M glycine and 0.2 M NaCl, pH 10.7) and fluorescence was measured at 465–360 nm using a microplate reader (Tecan reader infinite m200). The specific release was calculated in relation to the percentage of total β-hexosaminidase content that was obtained by lysing the cells with Triton X-100 (100%).

**Phl p 5-specific IgE, IgG1, IgG2a and IgA antibodies detection by ELISA**

For Phl p 5-specific antibody detection, maxisorp ELISA plates (Nunc, Thermo Fisher, Waltham, USA) were coated with Phl p 5 dilute in PBS at 2 µg/ml overnight at 4°C. After blocking and washing, plates were incubated with sera titrated using a starting dilution of 1:200, 1:50 or 1:3 for IgG1, IgG2a or IgA, respectively, following 1:2 dilution series for 2h at RT. Bound IgG1 was detected by an AP-conjugated rat anti-mouse IgG1 antibody and mouse IgG2a was detected using an AP-conjugated rat anti-mouse IgG2a antibody (all Southern Biotech, Birmingham, USA). Bound IgA was detected by an HRP-conjugated goat anti-mouse IgA antibody (Bio-Rad, Puchheim, Germany). After incubation for 1h at 37°C and 1h at 4°C, colorimetric detection with 10 mM 4-nitrophenyl phosphate (Sigma-Aldrich, St. Louis, MO, USA) was performed.

In order to enhance IgE detection, total IgG was captured from sera by using protein G sepharose beads (GE Healthcare), following the manufactures’ protocol. After IgG removal, sera titrated out starting from 1:20 were added to the plates previously coated with Phl p 5. Bound IgE was detected by an AP-conjugated rat anti-mouse IgE antibody (Southern Biotech, Birmingham, USA). After incubation for 1h at 37°C and 1h at 4°C, IgE detection was performed by 10 mM 4-nitrophenyl phosphate or TMB (Sigma, Wien, Austria). As a cutoff value the LOD (limit of detection) was used, which is calculated as three times the mean of the background. ELISA results are presented as endpoint titer, which was determined using the limit of quantification (10 times the standard deviation) adapted from a protocol described elsewhere ^3^. All measurements were performed in duplicates.

**Isolation of splenocytes and lymphocytes**

Spleens were aseptically removed immediately after mice sacrifice, disrupted and sedimented for 5 min in 1 ml minimal essential culture medium (MEM). Lysis of erythrocytes was performed by adding 5 ml ammonium chloride potassium (ACK) lysis buffer (150 mM NH_4_Cl, 10 mM KHCO_3_, 0.1 mM Na_2_EDTA in ddH_2_O, pH 7.2) for 5 min at RT. White blood cells were collected by centrifugation at 300 × g for 5 minutes at RT. Cells were resuspended in proliferation medium (MEM, 1% (v/v) heat-inactivated fetal calf serum, 2mM L-glutamine, 100U penicillin, and streptomycin/ml, 20mM Hepes, 1mM sodium pyruvate, 2 μM of 2-mercaptoethanol, and 1x non-essential amino acids) and used in the subsequent experiments. Skin-draining inguinal lymph nodes of 4get mice were collected in PBS. Lymph node surrounding fat tissue was removed and lymphocytes obtained using a tissue grinder.

**Splenocyte stimulation**

Splenocytes diluted at a density of 2 × 10^5^ in proliferation medium were added to a 96 well plate (BD Falcon) with Phl p 5 (20 µg/ml). The plates were incubated for 5 days at 37ºC, 5% CO_2_, and 95% relative humidity, and then submitted to flow cytometry analysis. To quantify cytokine levels in the supernatant of splenocytes after Phl p 5 stimulation, a multiplex bead-based flow cytometry analysis was performed using the mouse Th1/Th2/Th17/Th22 13plex FlowCytomix Multiplex kit (eBioscience) according to the manufacturer’s protocol. Measurements were performed on a Luminex MAGPIX instrument.

**Flow cytometry analysis**

After five days of stimulation, proliferating splenocytes were stained with anti-mouse CD25 (PE-conjugated), anti-mouse CD4 (V500-conjugated), and anti-mouse CTL4 (APC-conjugated) (all eBioscience, Vienna, Austria) at 4ºC for 30 minutes in the dark. For intracellular staining, a fixation/permeabilization buffer set was used according to the manufacturer's protocol (eBioscience, Vienna, Austria). Cells were washed with dPBS and incubated with fixation/permeabilization solution for 30 minutes at 4ºC in the dark. Cells were centrifuged at 1200 rpm for 5 min, washed twice with permeabilization solution and incubated with anti-mouse foxp3 (BV421-conjugated) and anti-mouse Ki-67 (PE/Cy7-conjugated) (Both BioLegend, San Diego, USA) for 30 minutes at 4ºC in the dark. Cells were washed twice with permeabilization solution and ressuspended in FACS buffer (1X PBS, 3mM EDTA). Fixable Viability Dye eFluor™ 780 (eBioscience) was used for live/dead discrimination. Doublet discrimination was performed by gating FSC width versus FSC height, and SSC height versus SSC area. Only living CD4+ splenocytes were used for further analysis. The gating strategy for Treg characterization is shown in Figure E3. Extracted lymphocytes from 4get mice were incubated with APC-conjugated anti-mouse CD4 antibody for 30 minutes at 4ºC in the dark (eBioscience, Vienna, Austria). Measurements were performed on a FACS Canto II flow cytometer (Becton, Dickinson and Company, Franklin Lakes, New Jersey, USA) and data analysis was performed using BD FACSDiva software (BD 78 Biosciences).

**ELISPOT**

For IFN-γ, IL-4 and IL-10 cytokines detection in splenocytes, 96 multiscreen filtration plates (Millipore) were activated with 70% ethanol for 10 minutes, washed 3 times with PBS, and coated with 2 μg/ml with anti-mouse IFN-γ (BioLegend), anti-mouse IL-4 (eBioscience), or anti-mouse IL-10 (BioLegend) overnight at 4°C in humidified chamber. Plates were washed 3 times with PBS and incubated for 2 hours with blocking solution (Proliferation medium supplemented with 5% fetal bovine serum) in humidified chamber at RT. Splenocytes were diluted at a density of 2 × 10^5^ in proliferation medium, added to each plate in the presence of Phl p 5 diluted at 25 μg/ml, and incubated for 48 h at 37°C and 5% CO_2_. After 3 washes with PBS supplemented with 0.1% Tween-20, IFN-γ, IL-4 and IL-10 (all BioLegend) detection biotin anti-mouse antibodies were added to the plates and held for 2h at RT in humidified chamber. After 3 washes, HRP-conjugated streptavidin was added to the plates and incubated for 1h at RT in humidified chamber. After 4 washes, TMB substrate (SeraCare Life Sciences) was added to each plate for 5 min prior to stopping by washing with ddH_2_O. Immunospots were counted using the color deconvolution plugin in ImageJ software.

**Online Repository references**

1. Oostingh GJ, Schmittner M, Ehart AK, Tischler U, Duschl A. A high-throughput screening method based on stably transformed human cells was used to determine the immunotoxic effects of fluoranthene and other PAHs. *Toxicol in Vitro.* 2008;22(5):1301-1310.

2. Vogel L, Luttkopf D, Hatahet L, Haustein D, Vieths S. Development of a functional in vitro assay as a novel tool for the standardization of allergen extracts in the human system. *Allergy.* 2005;60(8):1021-1028.

3. Frey A, Di Canzio J, Zurakowski D. A statistically defined endpoint titer determination method for immunoassays. *J Immunol Methods.* 1998;221(1-2):35-41.
