# Supplementary material for: TGFβ1 mimetic peptide modulates immune response to grass pollen allergens in mice
Source: Allergy. 2019 Dec 12;75(4):882–91. doi: 10.1111/all.14108 (PMC7217028; doi:10.1111/all.14108)
Supplement: Supplementary file 2 [file ALL-75-882-s002.pdf]

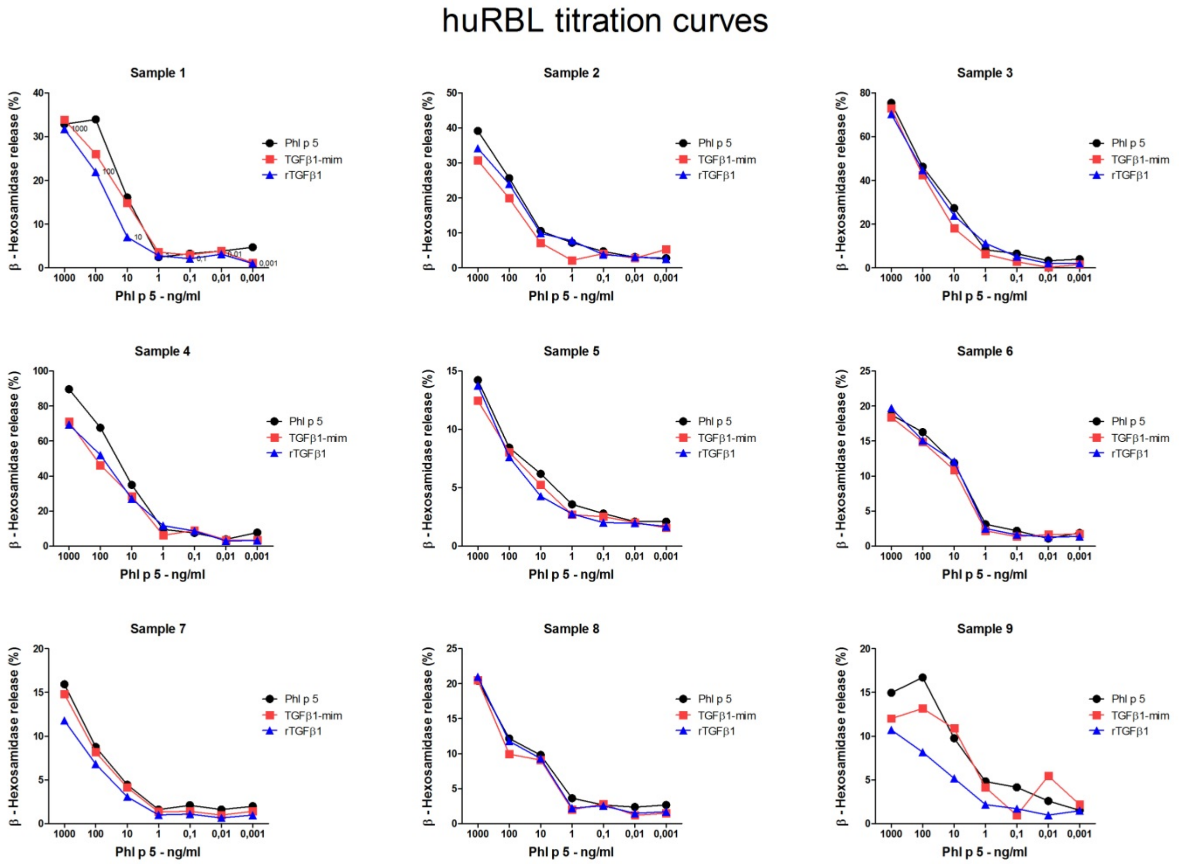

Figure E1. Titration curves of huRBL-2H3 cells encoding the human FcεRI receptor. Cells were sensitized overnight with sera from 9 different grass pollen allergic patients, treated for 24 h either with TGFβ1-mim peptide or recombinant TGFβ1, and then challenged for 1 h with the indicated concentrations of recombinant Phl p 5.
