# Supplementary material for: TGFβ1 mimetic peptide modulates immune response to grass pollen allergens in mice
Source: Allergy. 2019 Dec 12;75(4):882–91. doi: 10.1111/all.14108 (PMC7217028; doi:10.1111/all.14108)
Supplement: Supplementary file 3 [file ALL-75-882-s003.pdf]

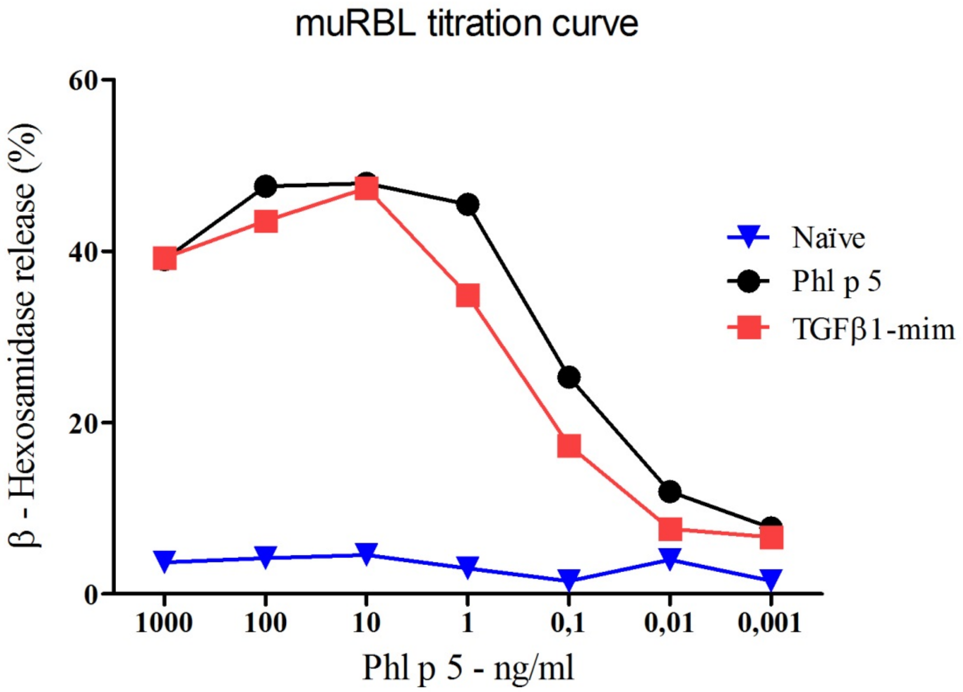

Figure E2. Titration curves of muRBL-2H3. Cells were sensitized overnight with pooled sera from the different immunization groups or naïve mice, and then challenged for 1 h with the indicated concentrations of recombinant Phl p 5. The optimal Phl p 5 concentration (1ng/ml) was used for maximal release assay with individual serum samples.
