# Supplementary material for: TGFβ1 mimetic peptide modulates immune response to grass pollen allergens in mice
Source: Allergy. 2019 Dec 12;75(4):882–91. doi: 10.1111/all.14108 (PMC7217028; doi:10.1111/all.14108)
Supplement: Supplementary file 4 [file ALL-75-882-s004.pdf]

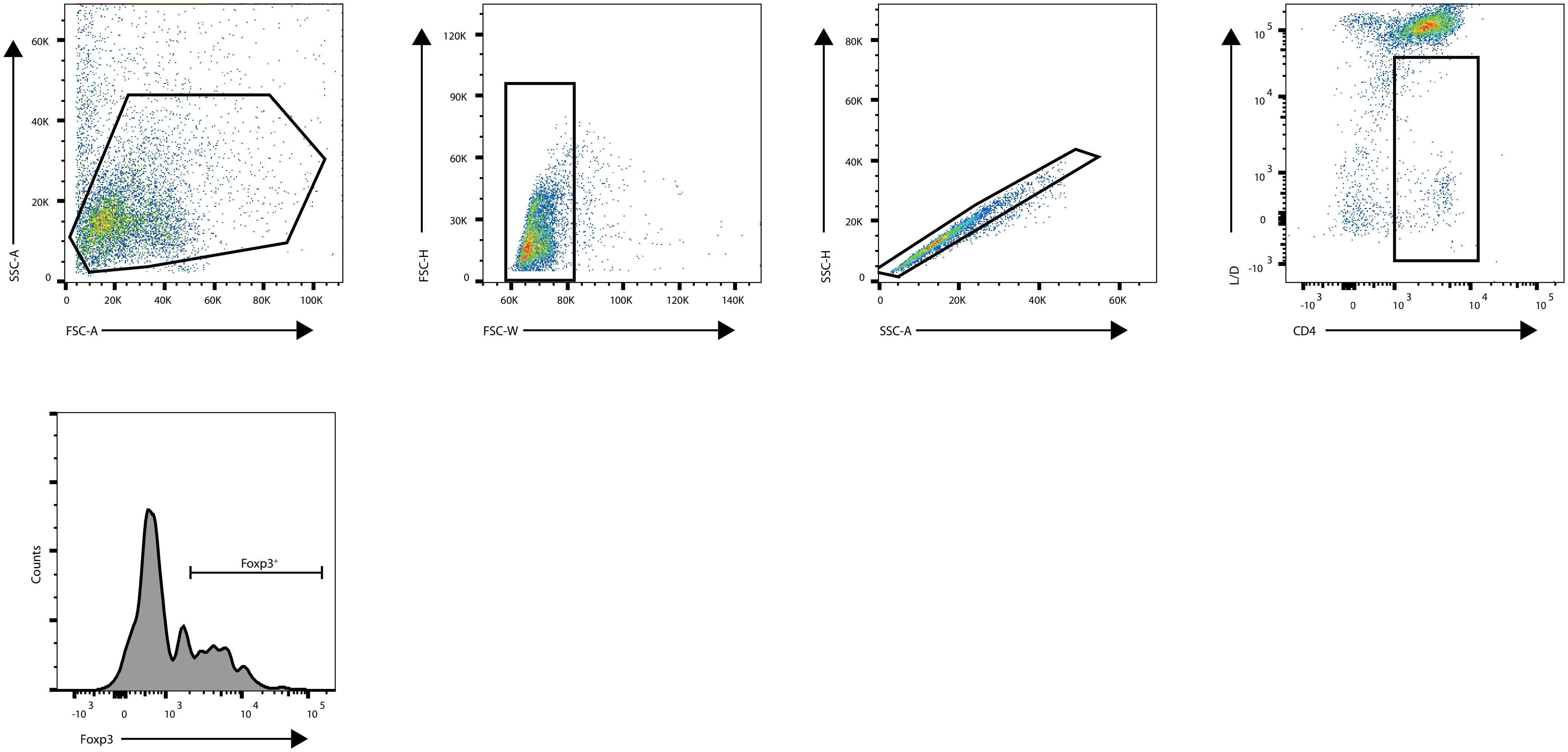

Figure E3. Gating strategy for identification of Treg cells. Splenocytes were gated based on scatter light (FSC, SSC) characteristics, followed by doublet discrimination (FSC width versus FSC height, and SSC height versus SSC area). Living CD4+ cells were gated (dot blot) and analyzed towards Foxp3 expression (histogram).
